# Vascular co-option in resistance to anti-angiogenic therapy

**DOI:** 10.3389/fonc.2023.1323350

**Published:** 2023-12-11

**Authors:** Domenico Ribatti, Tiziana Annese, Roberto Tamma

**Affiliations:** ^1^ Department of Translational Biomedicine and Neuroscience, University of Bari Medical School, Bari, Italy; ^2^ Department of Medicine and Surgery, Libera Università del Mediterraneo (LUM) Giuseppe Degennaro University, Bari, Italy

**Keywords:** angiogenesis, anti-angiogenesis, resistance, tumor growth, vascular co-option

## Abstract

Three different mechanisms of neovascularization have been described in tumor growth, including sprouting angiogenesis, intussusceptive microvascular growth and glomeruloid vascular proliferation. Tumors can also grow by means of alternative mechanisms including vascular co-option, vasculogenic mimicry, angiotropism, and recruitment of endothelial precursor cells. Vascular co-option occurs in tumors independently of sprouting angiogenesis and the non-angiogenic cancer cells are described as exploiting pre-existing vessels. Vascular co-option is more frequently observed in tumors of densely vascularized organs, including the brain, lung and liver, and vascular co-option represents one of the main mechanisms involved in metastasis, as occurs in liver and lung, and resistance to anti-angiogenic therapy. The aim of this review article is to analyze the role of vascular co-option as mechanism through which tumors develop resistance to anti-angiogenic conventional therapeutic approaches and how blocking co-option can suppress tumor growth.

## Angiogenesis and alternative mode of growth of tumor vasculature

**Figure 1:**
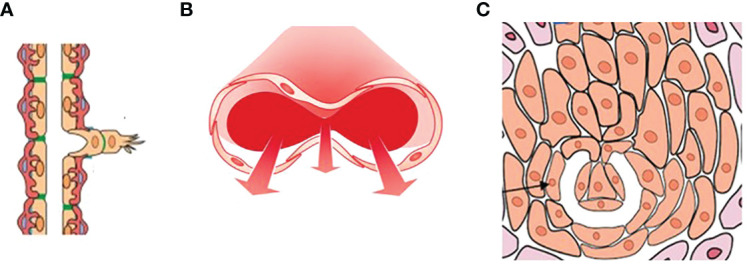
A drawing showing the three types of angiogenesis have been described in tumor growth: **(A)** sprouting angiogenesis, **(B)** intussusceptive microvascular growth (IMG), and **(C)** glomeruloid vascular proliferation. Sprouting angiogenesis involves formation and outgrowth of sprouts; IMG involves the formation of new vasculature where a pre-existing vessel splits in two; in glomeruloid vascular proliferation small glomeruloid bodies, so-called for their morphological resemblance with the renal glomeruli, are recognizable. [Reproduced from (5)].

Three types of angiogenesis have been described in tumor growth: sprouting angiogenesis in which vessel outgrowth from existing vessels is initiated by specialized endothelial cells termed tip cells (1, 2), intussusceptive microvascular growth (IMG), in which the vascular network expands by insertion of newly formed columns of interstitial tissue structures (tissue pillars) into the vascular lumen (3), and glomeruloid vascular proliferation, in which small glomeruloid bodies, so-called for their morphological resemblance with the renal glomeruli, are recognizable (4) (**Figure 1**).

Tumors can also grow without inducing angiogenesis, as occurs in vessel co-option or vascular co-option, vasculogenic mimicry, angiotropism, and recruitment of endothelial precursor cells (EPCs). In vasculogenic mimicry, first described in uveal melanoma (6) and subsequently in other cancers, tumor cells acquire an endothelial phenotype and form vessel-like networks. Vasculogenic mimicry can serve as a marker for tumor metastasis, a poor prognosis, worse survival, and the highest risk of cancer recurrence. Angiotropism (the pericytic-like location of tumor cells) is a marker of migration of melanoma and glioma tumor cells along the abluminal vascular surface (7). EPCs may be recruited from bone marrow mobilized by vascular endothelial growth factor A (VEGFA) or C-X-C motif chemokine 12 (CXCL12) released by tumor-infiltrating myeloid cells or cancer cells (8). However, most human tumors remain *in situ* without angiogenesis for a long time before they switch to an angiogenic phenotype (9).

The aim of this review article it to describe in detail through a retrospective analysis of the literature data the role of vascular co-option in the growth of primary and metastatic tumors. Moreover, the involvement of co-option in the development of resistance to conventional anti-angiogenic therapies and the possibility to overcome the resistance will be also described.

## Vascular co-option

**Figure 2:**
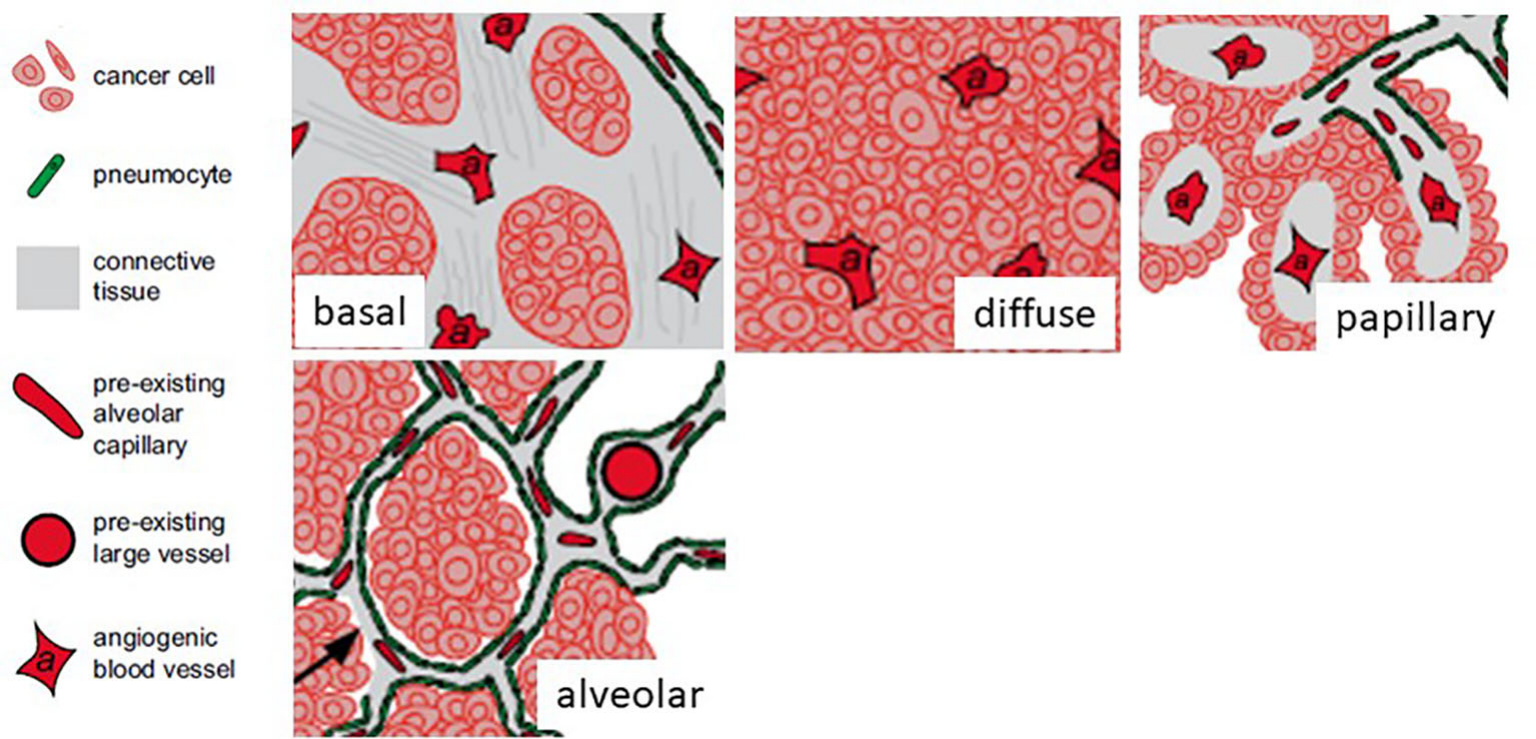
Four different histopathological growth patterns in non-small cell lung cancer. In three of these patterns (basal, diffuse, and papillary), the tumors are angiogenic, whereas in the fourth pattern (alveolar), the cancer cells grow in the alveolar air space of the lung and co-opt alveolar capillaries. [Modified from (11)].

In the first work on vascular co-option the non-angiogenic cancer cells were described as “exploiting” pre-existing vessels (10). Four different histopathological growth patterns have been described in non-small cell lung cancer. In three of these patterns (basal, diffuse, and papillary), the tumors are angiogenic, whereas in the fourth pattern (alveolar), the cancer cells grow in the alveolar air space of the lung and co-opt alveolar capillaries (10) (**Figure 2**). Vascular co-option, described in primary and secondary (metastatic) sites (**Table 1**) is defined as a process in which tumor cells interact with and exploit the pre-existing vasculature of the normal tissue in which they grow without the need for vascular proliferation (16). The pre-existing vasculature can be co-opted in two ways: tumor cells replace normal epithelial cells or penetrate the stroma surrounding the blood vessels (18). The host vasculature is incorporated by the growing tumor (19). In vascular co-option, cancer preserves the well-arranged vascular architecture of the normal tissue within the tumor, and tumors utilize alternative mechanisms besides angiogenesis to obtain nutrients for growth through local tumor invasion and proliferation along co-opted vessels. Cancer cells migrate along the pre-existing vessels and infiltrate tissues between co-opted vessels (16). By means of single-cell transcriptomic analysis, it has been demonstrated that co-opted endothelial cells and pericytes are characterized by similar transcriptomic signature of quiescent healthy endothelial cells and different from tumor endothelial cell signature recognizable in angiogenic tumors, characterized by tip, proliferating, and immature endothelial cells (20). Moreover, different cell types involved in co-option, including an invasive cancer cell subtype and a M1-like macrophage subtype have been identified (20).

**Table 1:** Evidence for vascular co-option in human cancers (References between brackets).

**Brain**	• Primary brain cancer (12) • Metastasis derived from breast cancer, colorectal cancer, lung cancer, melanoma (13)
**Lymph node**	• Metastasis derived from breast cancer, colorectal cancer, head and neck cancer (14)
**Lung**	• Primary lung cancer (10) • Metastasis derived from breast cancer, colorectal cancer, renal cell carcinoma (15)
**Skin**	• Metastasis derived from breast cancer (16)
**Liver**	• Primary liver cancer (16) • Metastasis derived from breast cancer, colorectal cancer (17)

Some tumors grow by exploiting the host vessels and initiating pre-existing blood-vessel-dependent tumor growth. These vessels then regress owing to endothelial cell apoptosis, mediated by angiopoietin- 2 (Ang-2), whereas angiogenesis occurs at the periphery of the growing tumor by the interaction of VEGF and Ang-2 (21). The co-opted vessels trigger an apoptotic cascade, probably by autocrine induction of Ang-2, followed by vessel regression resulting in tumor death. However, successful tumors overcome this vessel regression by initiating neoangiogenesis (22). Vascular co-option is more frequently observed in tumors of densely vascularized organs, including the brain, lung, and liver, where the primary tumor cells co-opt the adjacent quiescent blood vessels of the host tissue. In the brain, cancer cells can adhere to the abluminal surface of the brain vessels and grow around them or can infiltrate the brain parenchyma and migrate between brain vessels. Vessel co-option has been reported in both low- and high-grade gliomas (12). In advanced hepatocellular carcinoma, vascular co-option may interest vessels of the portal tract or liver sinusoidal vessels (16). The lymph node is also involved in nonangiogenic growth by vascular co-option (14). 

## Vascular co-option and metastases

Tumor cells have immediate access to blood vessels, such as when they metastasize to or are implanted within a vascularized tissue, co-opt, and often grow as cuffs around adjacent existing vessels (21). The ability of cancer cells to co-opt the vessels initiates the process through a few of them will intravasate, starting the metastatic process (23). Invasive cancer cells express Wnt7b, that promotes metastasis and vascular co-option in an experimental model of glioma (24).

The blood vessels at the interface between the tumor and surrounding tissue can be co-opted through the replacement of epithelial cells by tumor cells and/or the invasion of tumor cells into the stroma surrounding the blood vessels (25, 26). Many patients with non-angiogenic *vs*. angiogenic primary lung tumors developed distant lung metastases and vessel-co-option was associated with decreased overall survival, and a 3D-tumor reconstruction showed that the blood vessel immunostaining resembled a normal lung (27–29). In breast cancer lung metastases, non-angiogenic growth was present in 19% of the lung metastases (30).

Melanoma cells migrate extra vascularly in the brain metastasis along the basement membrane of the blood vessels surrounding the tumor, constituting an alternative mechanism of tumor spreading instead of intravascular dissemination (angiotropism or pericytic mimicry) (7, 31, 32). Vessel co-option occurs in other secondary brain tumors, including brain metastases of breast, colorectal, and lung cancer (13). Carcinomas that metastasize to the lung, including breast, colorectal, gastric, pancreatic, and renal cancer, may be characterized by vascular co-option (10, 15, 30, 33). Vessel co-option can occur in breast cancer metastases to many organs, including the lungs, liver, lymph nodes, skin, and brain (10, 17, 30, 34, 35).

## Experimental models used for the study of vascular co-option and their impact on therapy response

Different animal models have been established to study vascular co-option. By means of intracranial transplantation it has been demonstrated that different tumor cell lines are able to co-opt pre-existing brain vessels (21). Anti-VEGF and anti-VEGF receptor 2 (VEGFR2) treatments increased the number of co-opted brain vessels after injection of human glioblastoma cell lines into nude rat striatum (36, 37). By means of intracardial transplantation into the left cardiac ventricle of anesthetized mice it has been demonstrated that serpins promote cancer cell survival and vascular co-option in brain metastasis (21). Through direct injection of tumor cells into the internal carotid artery of anesthetized mice, it has been demonstrated that VEGFA favors brain metastasis of different human melanoma cell lines to grow by means of vascular co-option without sprouting angiogenesis (38). Moreover, anti-angiogenic therapy of cerebral melanoma metastases results in sustained tumor progression via vascular co-option (39). By means of intravenous injection via jugular vein, it has been demonstrated that lung metastases co-opted the lung microvasculature (20). Moreover, by means of the same experimental model, it was shown that vascular co-option mediates resistance to anti-angiogenic therapy in lung metastasis (15). Using orthotopic injection of human hepatocellular cell lines into the livers, it has been demonstrated that vascular co-option is involved in acquired resistance to anti-angiogenic therapy (40). By means of zebrafish and chick embryo chorioallantoic membrane (CAM) assays it has been shown that connexins are involved in metastatic breast cancer and melanoma brain colonization through vascular co-option (41).

## Resistance to anti-angiogenic therapies

When VEGF-targeted therapies are discontinued, the tumor vasculature is rapidly re-established (42). Resistance to anti-angiogenic therapies can be intrinsic, when it is observed at the beginning of the treatment due to inefficacy of treatment or acquired, i.e., that it affects the relapsing disease after an initial response to therapy (43, 44). The main mechanisms of resistance involve: 1) Vascular co-option (45); vasculogenic mimicry; IMG (a switch from sprouting to IMG represents an adaptive response to treatment with anti-angiogenic compounds to restore the hemodynamic and structural properties of the vasculature-enhancing tumor drug delivery and sensitivity to treatments (46). 2) Hypoxia that increases the expression of pro-angiogenic factors. The relationship between hypoxia, VEGF, and angiogenesis has significant implications for response to therapy, and hypoxic areas are refractory to chemotherapy and radiotherapy. VEGF blockade aggravates hypoxia that, in turn, upregulates the production of angiogenic factors or increases tumor invasiveness (43, 47). 3) Redundancy of the angiogenic signals, and activation of alternative signaling pathways [e.g., platelet derived growth factor/platelet derived growth factor receptor (PDGF/PDGFR); fibroblast growth factor/fibroblast growth factor receptor (FGF/FGFR); Ang2/Tie 2] (48). 4) Upregulation of other pro-angiogenic factors, including FGF2 and PDGF (48). 5) Vascular heterogeneity, and EPCs recruitment.

In metastatic colorectal cancer patients, bevacizumab treatment was associated with increased plasma levels of placental growth factor (PlGF), FGF, and PDGF prior to and along disease progression (49). Colorectal patients with poor response to bevacizumab had increased Ang2 serum levels, and VEGF and Ang2 blockade delayed tumor growth, normalized tumor vasculature, and increased survival (50, 51).

## Resistance and co-option

In patients with metastatic colorectal cancer treated with neoadjuvant bevacizumab, and then underwent complete resection of the metastases, a higher number of nonangiogenic lesions was found in the viable tissue of the poor responder while prevalently angiogenic metastases were seen among the good responders (52). In two cohorts of breast cancer patients with liver metastases, one treated with bevacizumab and chemotherapy and one with chemotherapy alone prior to metastases resection, all of the metastatic lesions examined, were nonangiogenic (52). In colorectal cancer liver metastases, from three cohorts, one not receiving any neoadjuvant, a second receiving neoadjuvant chemotherapy, and a third treated with chemotherapy plus bevacizumab, upon treatment with chemotherapy and bevacizumab, the nonangiogenic lesions showed no or minimal necrosis while the angiogenic tumors had no more than 2% of viable cells (53). A similar pattern was seen in the patients treated with chemotherapy alone, the only difference being that in the angiogenic tumors, the percentage of viable tissue was higher. Untreated nonangiogenic metastases were comparable to the treated ones, while untreated angiogenic had a more variable amount of necrosis, supporting the hypothesis that nonangiogenic tumors are not only more resistant to bevacizumab but also to the neoadjuvant chemotherapy used (53).

No differences in intratumor vascularization were found in node metastases from colorectal cancer patients treated or not treated with bevacizumab (34). Antiangiogenic treatment of primary central nervous system tumors has been used for several years but, again, the results have not lived up the expectations (54). Vascular co-option mediates resistance to anti-angiogenic therapy in liver metastases (52), and in an experimental model of hepatocellular carcinoma, a switch to vascular co-option has been described as a mechanism of resistance to treatment with tyrosine kinase inhibitor sorafenib, where over time, tumor cells become more invasive which promotes co-option of liver vessels in the face of angiogenesis blockade (55).

Histological examination of patients who died after receiving treatment with cediranib, an inhibitor of VEGFR2 tyrosine kinases (56) or bevacizumab regimen (57) showed that the glioma cells were growing around preexisting vessels in a nonangiogenic fashion. Vascular co-option and progression in the absence of angiogenesis in human brain tumor samples, surgical and autoptic, have been illustrated (26, 54). Breast cancer patients treated with bevacizumab showed, by dynamic contrast-enhanced magnetic resonance imaging (DCE-MRI), three response patterns. In the first one, a decrease in K-trans values over the extent of the tumor was demonstrated, that is, decreased vascular permeability and/or vascular surface area, while the second was characterized by extensive necrosis. These two patterns indicate a response to anti-angiogenic treatment. In the third one, instead, no changes were seen, and the authors conclude that, in these lesions, the vessels were independent of VEGF. Co-option of pre-existing vascular beds in adipose tissue controls tumor growth rates and angiogenesis (58).

Metastatic lesions in stage III melanoma patients treated with bevacizumab had a mature vessel morphology and phenotype in contrast with the newly formed vessels in the relapsing disease from the patients not receiving bevacizumab (59). Lung metastases of renal cell carcinoma (RCC) can escape anti-angiogenic treatment with cediranib and gefitinib by switching phenotype and progressing in an angiogenesis-independent fashion exploiting preexisting vessel (60). Treatment of glioma with a monoclonal antibody against VEGFR2 induced co-option in quiescent cerebral vessels (37). In glioblastoma biopsies taken after antiangiogenic treatment, a pattern of infiltration around the normal brain vessels is present (26). Nonangiogenic growth and spread in the lung have also been linked to resistance to surgical treatment (61). In nonangiogenic carcinoma growing in the lung, neoplastic cells can spread by moving from one alveolar cavity to the other through the septa pores (61). Co-option of liver vessels has been demonstrated in acquired sorafenib resistance in hepatocellular carcinoma (40).

## Combination therapy to overcome resistance to anti-angiogenic therapy

Dual inhibition of VEGF/PDGFR improves survival in glioblastoma (62). Bevacizumab-resistant patients exhibit an up-regulation of c-MET expression (63). The concomitant exposure to hepatocyte growth factor (HGF)/c-MET inhibitors and sunitinib abrogated angiogenesis and tumor growth (64). Application of brivanib, a dual inhibitor of FGF and VEGF pathways, in bevacizumab-resistant tumors induced an increased overall survival in breast cancer (65). Aflibercept, a dual inhibitor of VEGF and PlGF, and bevacizumab are effective in patient-derived xenograft models of colorectal cancer (66). Lenvatinib, a multiple receptor tyrosine kinase inhibitor of VEGFRs 1-3, FGFRs 1-4, KIT, PDGF receptor alpha (PDGFRα), and RET (rearranged during transfection) exerts antiproliferative and anti-angiogenic effects in hepatocellular carcinoma (67). Otherwise, both dovitinib and nintedanib (VEGF, FGF, and PDGF receptor tyrosine kinase inhibitors) are ineffective (68, 69).

## Combination therapy to overcome resistance and vascular co-option

Combinatorial treatment approaches integrating anti-angiogenic therapy with blockade of vascular co-option can be considered. Anti-angiogenic therapy via VEGFR2 blockade reduced intracerebral glioblastoma growth but caused an increase in tumor migration with a vascular co-option pattern. The increase in cancer cell migration may be inhibited by combined treatment with VEGFR2 and epidermal growth factor receptor (EGFR) antibodies, as demonstrated by a decreased *in vitro* migration of glioblastoma cells (70).

A single agent vanucizumab, a bispecific anti-Ang2/anti-VEGFA antibody, was tested in a phase I study in adult patients with advanced solid tumors (71). Dual inhibition of Ang2 and VEGFRs normalizes tumor vasculature and prolongs survival in glioblastoma (51). Stimulation of Ang-2 expression in endothelial cells together with inhibition of VEGF signaling may inhibit vascular cooption. Signaling of Ang-2 through its receptor Tie-2 can cause sprouting angiogenesis if VEGF levels in the tumor microenvironment are high. If VEGF levels are low, Ang-2/Tie-2 signaling leads to the regression of co-opted vessels (72–74).

Inhibition of hypoxia-related macrophages and neutrophils may inhibit vascular co-option. Accumulation of lysyl oxidase like 4 (LOXL-4)-expressing neutrophils in colorectal lung cancer metastases resistant to anti-angiogenic therapy where vascular co-option is the dominant pattern of vascularization (75). M1 tumor associated macrophages (TAMs) are highly detected in vascular co-option-dependent tumors (20). Treatment with bevacizumab caused a metabolic shift toward glycolysis, promoting the vascular co-option pro-invasive phenotype in glioblastoma (76).

## Concluding remarks

Many cancers can co-opt the pre-existing vasculature and, in this manner, facilitate tumor growth *in situ* and at distance. The existence of non-angiogenic tumors allows to predict that these tumors are non-sensitive to anti-angiogenic treatments (77). Several preclinical studies support the concept that vessel co-option can mediate intrinsic and acquired resistance to anti-angiogenic therapy, and co-option may in part explain the inefficacy of anti-angiogenic cancer therapies. In this context, blocking or inhibiting co-option might be considered as an innovative strategy in the inhibition of the growth of certain tumors, which adopt this modality of expansion, suggesting that therapeutic strategies targeting both angiogenesis and co-option might be more efficacious than targeting angiogenesis alone (73). It will be also important to consider the role of vascular co-option in the modulation of the host immune response to tumor and therefore also the efficacy of immunotherapy in the treatment of tumors (78). In this context, immune checkpoint-inhibitor treatment might induce an immune response against the co-opting cancer cells and synergize with anti-angiogenic agents (79, 80).

## Author contributions

RT: Writing – review & editing. DR: Writing – original draft. TA: Writing – review & editing.
